# Treatment of neonatal infections: a multi-country analysis of health system bottlenecks and potential solutions

**DOI:** 10.1186/1471-2393-15-S2-S6

**Published:** 2015-09-11

**Authors:** Aline Simen-Kapeu, Anna C Seale, Steve Wall, Christabel Nyange, Shamim A Qazi, Sarah G Moxon, Mark Young, Grace Liu, Gary L Darmstadt, Kim E Dickson, Joy E Lawn

**Affiliations:** 1 Health Section, Programme Division, UNICEF Headquarters, 3 United Nations Plaza, New York, NY 10017, USA; 2 University College London Department of Infectious Diseases Informatics, UCL Institute for Health Informatics, Farr Institute, 222 Euston Road, London NW1 2DA, UK; 3 Saving Newborn Lives, Save the Children, 2000 L Street NW, Suite 500, Washington, DC 20036, USA; 4 Ross University Medical School, 2300 SW 145th Avenue, Miramar, FL 33027, USA; 5 Department of Maternal, Newborn, Child and Adolescent Health, World Health Organization, Avenue Appia 20, 1211 Geneva 27, Switzerland; 6 Maternal, Adolescent, Reproductive and Child Health (MARCH) Centre, London School of Hygiene and Tropical Medicine, London, WC1E 7HT, UK; 7 Save the Children Federation, Inc., 501 Kings Highway East, Suite 400, Fairfield, CT 06825, USA; 8 Department of Infectious Disease Epidemiology, London School of Hygiene and Tropical Medicine, London, WC1E 7HT, UK; 9 Department of Pediatrics, Stanford University School of Medicine, Stanford, CA 94305 USA

**Keywords:** Neonatal infections, sepsis, antibiotics, bottlenecks, barriers, solutions, primary health care, community-based health care

## Abstract

**Background:**

Around one-third of the world's 2.8 million neonatal deaths are caused by infections. Most of these deaths are preventable, but occur due to delays in care-seeking, and access to effective antibiotic treatment with supportive care. Understanding variation in health system bottlenecks to scale-up of case management of neonatal infections and identifying solutions is essential to reduce mortality, and also morbidity.

**Methods:**

A standardised bottleneck analysis tool was applied in 12 countries in Africa and Asia as part of the development of the *Every Newborn *Action Plan. Country workshops involved technical experts to complete a survey tool, to grade health system "bottlenecks" hindering scale up of maternal-newborn intervention packages. Quantitative and qualitative methods were used to analyse the data, combined with literature review, to present priority bottlenecks and synthesise actions to improve case management of newborn infections.

**Results:**

For neonatal infections, the health system building blocks most frequently graded as major or significant bottlenecks, irrespective of mortality context and geographical region, were health workforce (11 out of 12 countries), and community ownership and partnership (11 out of 12 countries). Lack of data to inform decision making, and limited funding to increase access to quality neonatal care were also major challenges.

**Conclusions:**

Rapid recognition of possible serious bacterial infection and access to care is essential. Inpatient hospital care remains the first line of treatment for neonatal infections. In situations where referral is not possible, the use of simplified antibiotic regimens for outpatient management for non-critically ill young infants has recently been reported in large clinical trials; WHO is developing a guideline to treat this group of young infants. Improving quality of care through more investment in the health workforce at all levels of care is critical, in addition to ensuring development and dissemination of national guidelines. Improved information systems are needed to track coverage and adequately manage drug supply logistics for improved health outcomes. It is important to increase community ownership and partnership, for example through involvement of community groups.

## Background

Neonatal (0-28 days) deaths account for an estimated 44% of deaths in children under age 5 years [1]. Around one-third of these, (640,000 in 2013) are caused by neonatal infections, including the clinical syndromes of sepsis, meningitis and pneumonia [1]. In total, there were an estimated 6.1 million cases of possible serious bacterial infection (PSBI) in South Asia, sub-Saharan Africa in 2012 [2]. As well as mortality, these neonatal infections cause impairment and disability [2,3]. Newborns most at risk are preterm and/or low birth weight [4,5]. Prevention measures include immunisation [6,7], anti-sepsis [8-11], and exclusive breastfeeding [12,13]. For those who do become sick, timely detection of newborn illness with appropriate case management could prevent an estimated 84% of neonatal infection deaths [14,15].

**Figure 1:**
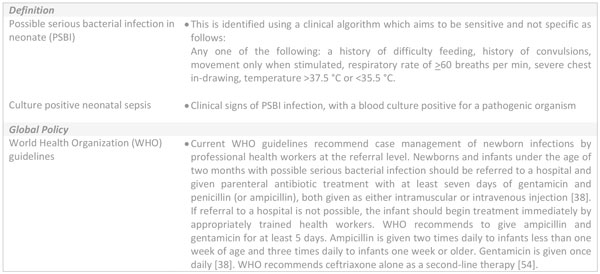
**Clinical algorithm for neonatal infection and World Health Organization recommended treatment**. PSBI: possible serious bacterial infection; WHO: World Health Organization.

**Figure 2:**
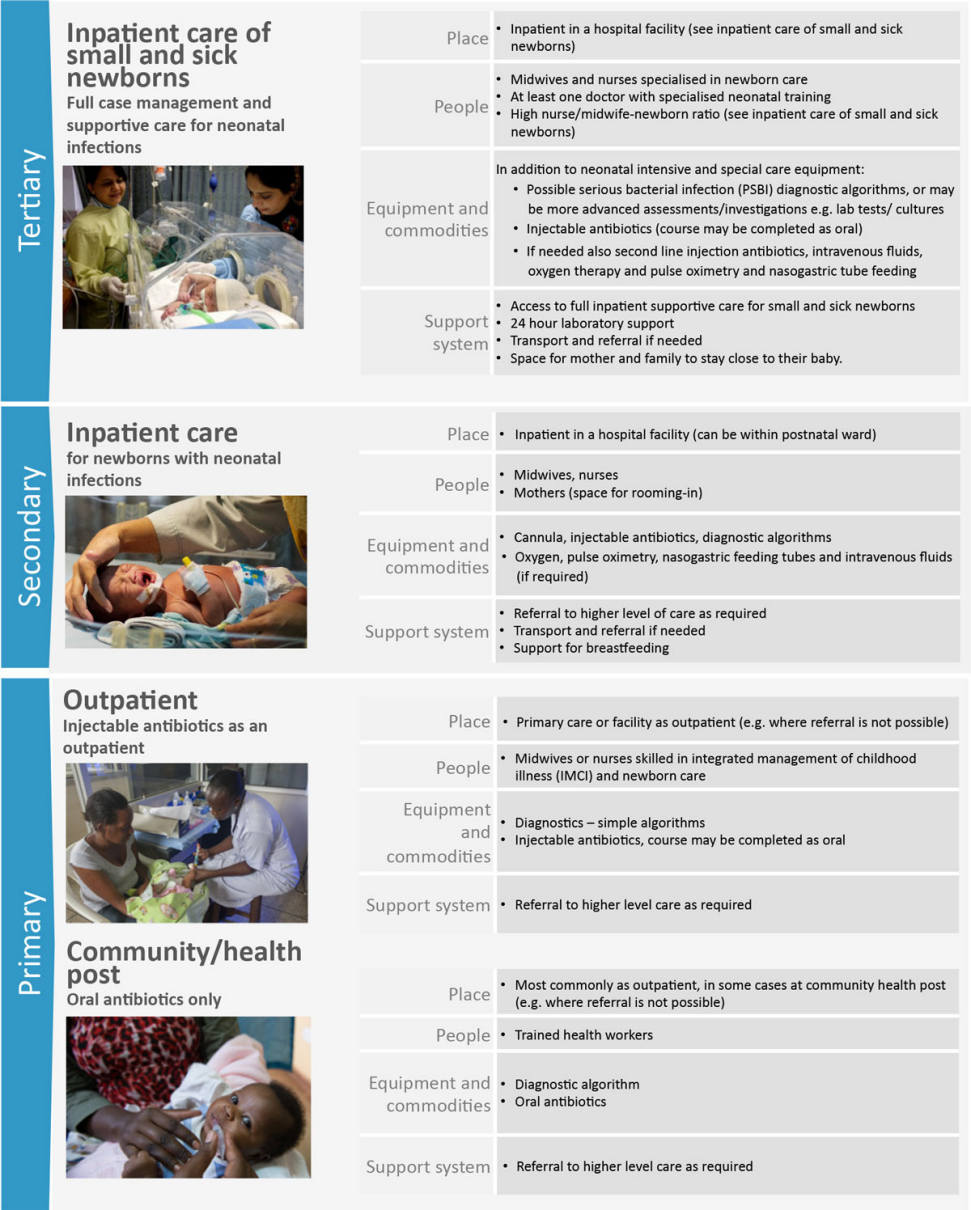
**Treatment of neonatal infections, showing health system requirements by level care**. Inpatient care of small and sick newborns image source: Ayesha Vellani/Save the Children. Inpatient care image source: Syane Luntungan/Jhpiego. Outpatient care image source: Ian P. Hurley/Save the Children. Community/health post care: Hannah Maule-ffinch/Save the Children. PSBI: possible serious bacterial infection; IMCS: integrated management of childhood illness.

Newborns with infections can rapidly deteriorate and prompt identification and treatment are needed. Clinical algorithms are used to guide initiation of treatment, as part of the Integrated Management of Childhood Illness (IMCI) (Figure 1). Inpatient hospital care is the cornerstone of management of severe neonatal infection (Figure 2) [16]. WHO guidelines recommend sick newborns identified in the community are referred and admitted to a health facility for supportive care and treatment [17-19]; however, referral is not always possible. To improve access to care, simplified antibiotics for non-critically ill newborns can be safely provided by trained and supervised health workers as outpatients, if referral is not possible, as reported in recent clinical trials [6-8]. WHO is expected to soon release guideline for management of possible serious bacterial infection (PSBI) where referral is not possible.

Most critical, and most challenging, is transforming health systems to overcome barriers to improve treatment of newborn infections [9]. There are many preventable deaths, due to delays in diagnosis [10], care seeking, and treatment [11]. Healthcare constraints likely include a lack of trained health workers, and limited financial resources, as well as socio-cultural issues (reducing access to care) [20,21]. Health systems research aiming to identify bottlenecks to delivery, affordability, and sustainability of high-impact interventions to reduce mortality from newborn infections was recently highlighted as a research priority [12].

This paper is part of a nine paper series on quality maternal and newborn care and aims to describe intervention and context-specific health system bottlenecks, with potential solutions, for improved case management of neonatal infections. Care of small and sick newborns is detailed elsewhere in this series [22,23]. Specifically, the objectives of this paper are to:

1. Use a 12-country analysis to explore health system bottlenecks affecting the treatment of neonatal infections.

2. Present solutions to overcome the most significant bottlenecks including learning from the 12-country analyses, literature review and programme experience.

3. Discuss policy and programmatic implications and propose priority actions for the treatment of neonatal infections.

Results will guide sharpening of health programme priorities and policy and strengthen implementation of the *Every Newborn *Action Plan to end preventable newborn deaths by 2030 [13].

## Methods

This study used quantitative and qualitative research methods to collect information, assess health system bottlenecks and identify solutions to the scale up of neonatal infection case management. Other maternal and newborn care interventions were assessed as part of this process and are described elsewhere in the series. The participating countries were Afghanistan, Cameroon, Democratic Republic of Congo (DRC), Kenya, Malawi, Nigeria, Uganda, Bangladesh, India, Nepal, Pakistan and Vietnam, expanding on a smaller subset as published in The Lancet *Every Newborn *series [9].

### Data collection

The maternal-newborn bottleneck analysis tool (see Additional file 1) was developed to assist countries in the identification of bottlenecks to the scale up and provision of nine maternal and newborn health interventions across the seven health system building blocks, as described previously [9,16]. The tool was utilised during a series of national consultations supported by the global *Every Newborn *Steering Group between 1^st ^July and 31^st ^December 2013. The workshops for each country included participants from national Ministries of Health, United Nations agencies, the private sector, non-governmental organisations, professional bodies, academia, bilateral agencies and other stakeholders. For each workshop, a facilitator, orientated on the tool, coordinated the process and guided groups to reach consensus on the specific bottlenecks for each health system building block.

Tracer interventions were defined for each package of interventions to focus the workshop discussion. The use of injectable antibiotics was selected as a tracer indicator for quality care provided to newborns with neonatal infection.

### Data analysis methods

**Figure 3:**
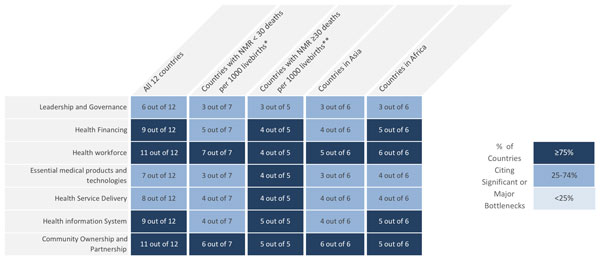
**Very major or significant health system bottlenecks for treatment of neonatal infections**. NMR: Neonatal Mortality Rate. *Cameroon, Kenya, Malawi, Uganda, Bangladesh, Nepal, Vietnam. **Democratic Republic of Congo, Nigeria, Afghanistan, India, Pakistan. See Additional file 2 for more details.

**Figure 4:**
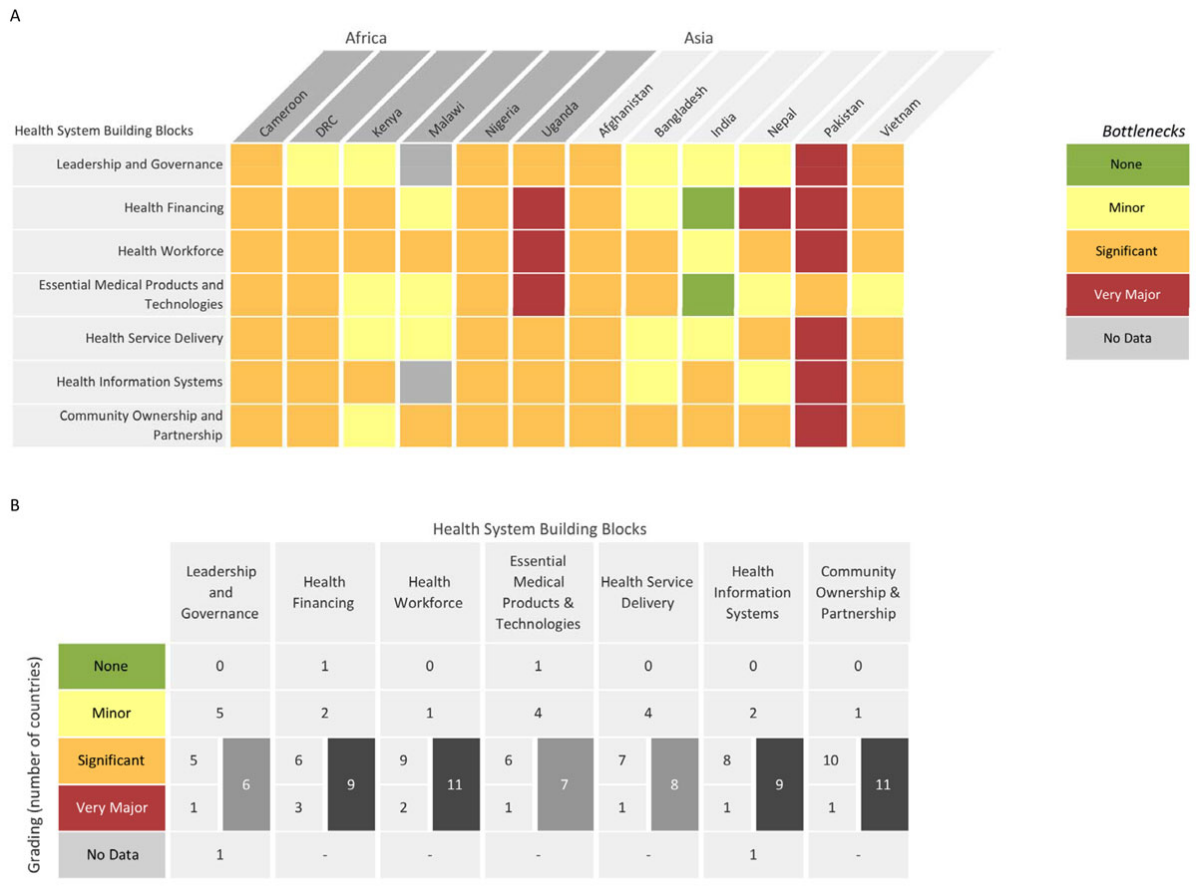
**Individual country grading of health system bottlenecks for treatment of neonatal infections**. Part A: Heat map showing individual country grading of health system bottlenecks for treatment of neonatal infections. Part B: Table showing total number of countries grading significant or major for calculating priority building blocks. DRC: Democratic Republic of the Congo.

Data received from each country were reviewed, consolidated, and categorised by the *Every Newborn *study group as described below. The graded health system building blocks were converted into heat maps (Figures 3 and 4). Bottlenecks for each health system building block were graded using one of the following options: not a bottleneck (=1), minor bottleneck (=2), significant bottleneck (=3), or very major bottleneck (=4). The number of countries from which workshop participants categorised health system bottlenecks as significant or very major are presented by mortality context (Neonatal Mortality Rate (NMR) <30 deaths per 1000 live births and NMR ≥30 deaths per 1000 live births) and region (Africa or Asia). Grading of health system bottlenecks by individual countries was also described.

Context specific solutions to overcome challenges were categorised into thematic areas and linked to specific bottlenecks. A literature review was undertaken to identify further case studies and evidence-based solutions for each defined thematic area. For a detailed description of the steps taken to analyse the intervention specific bottlenecks, refer to the overview paper [16].

## Results

Twelve countries were included in this exercise: Afghanistan, Cameroon, Democratic Republic of Congo (DRC), Kenya, Malawi, Nigeria, Uganda, Bangladesh, Nepal and Vietnam returned national level responses. Pakistan provided subnational data from all provinces apart from two territories which were Gilgit-Baltistan and Azad Jammu and Kashmir. India returned subnational data from three states: Andhra Pradesh, Odisha and Rajasthan (see Additional file 2). Data on suggested strategies and solutions were missing for Uganda and two provinces in Pakistan. However, bottleneck data from these countries were available and included in the analysis.

Overall, the bottlenecks rated by country teams as very major or significant health system bottlenecks (≥75% of countries) were as follows: health workforce (11 out of 12 countries), community ownership and partnership (11 out of 12 countries), health financing (9 out of 12 countries) and health management information systems (9 out of 12 countries) (Figure 3).

Minor regional differences were observed in the grading; very major or significant bottlenecks related to health financing and health management information system were reported by 5 out of 6 country teams from Africa and 4 out of 6 country teams from Asia. More notable differences were identified by mortality context. For countries with high NMR (≥30 deaths per 1000 live births), all the health system building blocks except for leadership and governance were cited as very major or significant bottlenecks by at least 4 out of 5 country teams (≥75% of countries) (Figure 3).

Common bottlenecks to optimal management of neonatal infections (reported by at least 3 countries) and their potential solutions are described below (summarised in Table 1 and Table 2) for the seven health system building block categories.

**Table 1:** Summary of solution themes and proposed actions for treatment of neonatal infections.

Health system building block	Solution themes	Proposed actions
Leadership and governance	*Policy/guidelines review, harmonisation and dissemination* *Task shifting policy* *Harmonisation and dissemination of guidelines*	• Review / formulation of policies and strategies: integration of neonatal care within integrated management of childhood illnesses (IMCI) and scale up of this at primary care level; • Shifting care and treatment at the outpatient level where referral is not possible- Expansion/implementation of community-based MNH programmes • Harmonise guidelines for management of neonatal infections at all level of care: engage professional bodies and private health care institutions • Ensure effective dissemination of guidelines (workshops, websites)

Health financing	*Funding for newborn health* *National insurance schemes expansion*	• Increase budget allocated for newborn health: ensure adequate resources for trainings of health workers, laboratory services, and commodities including injectable antibiotics • Expand health insurance schemes to address out-of-pocket payments: expansion of community-based health insurance schemes (e.g.: inclusion of fees required at teaching hospitals and for laboratory tests into insurance schemes

Health workforce	*Local recruitment of health workers* *Institutionalisation of incentives* *Competency-based skilled based pre-service and in-service training*	• Recruit local staff residing in the community to expand the availability of health care workers in all areas • Motivate health providers through institutionalisation of incentives to improve their retention in rural and hard to reach areas such as improved welfare packages or wages, hardship allowances, pay for performance • Conduct large scale pre-service and in-service trainings of health care workers in newborn care including management of neonatal infections; enhance the quality of trainings provided through supervision, mentoring and certification systems; involve medical colleges and institutions; train community-based health care workers in newborn care for home visits and identification of sick young infants

Essential medical products and technologies	*Procurement and supply chain management system* *Quality control mechanisms*	• Strengthen the national procurement and supply system: Forecast adequate amount of injectable antibiotics according to the needs for treatment and based on the buffer to be kept; Streamline the procurement process including fast tracking of essential commodities; Special equipment like, Continuous positive airway pressure, portable x-ray, and arterial blood gas analyser should made available for tertiary care centres; • Establish quality control mechanisms: Auditing of medical stores; facility assessments of those reporting frequent stock-outs; assessment of the quality of antibiotics in the bidding process; ensure the delivery of quality products; Develop an electronic logistic management system

**Table 2:** Summary of solution themes and proposed actions for treatment of neonatal infections (continued).

Health system building block	Solution themes	Proposed actions
Health service delivery	*Mobile outreach MNH services/ post-discharge counselling* *Community/home-based maternal and newborn care* *Two way referral system for sick newborns* *Quality assurance/ improvement mechanisms*	• Ensure post-discharge counselling on danger signs for newborns; • Scale-up newborn care at the lower level of care: Strengthen the provision of newborn care, especially sepsis case management at outpatient/primary health centre level where referral is not possible, institutionalise home visits / domiciliary care by trained personnel; Expand mobile outreach MNH services to make newborn services closer to the community • Strengthen two way referral system for sick newborns; • Establish quality assurance mechanisms: a quality assurance cell at the state/national level with regular quality assessments with emphasis on supportive supervision and mentoring by medical colleges and private hospitals; national scale-up of clinical audits and perinatal death reviews; Periodic critical review of appropriate management of newborn infections; Regular review and dissemination of quality of care check lists

Health information system	*Electronic reporting systems* *Processes and evidence-based decision -making*	• Develop electronic reporting system: software apps for record keeping on newborn interventions including neonatal sepsis management and establishing linkages from facility to community • Strengthen data collection and reporting for newborn care: disaggregate the data in the health management information system to include newborn health interventions, especially management of severe neonatal infections; add community data and postnatal consultations; develop neonatal registers; and set up a monitoring system for hospital infection prevention • Establish review processes; analyse and use data on management of neonatal infections to inform performance review meetings and for quality improvement processes • Enhance research in newborn care: conduct bacterial surveillance and antibiotics resistance studies; conduct newborn survival analysis to inform programme managers

Community ownership and participation	*Development/review of local IEC materials* *Fee exemption for newborn care* *Community-based education for behaviour change* *Male involvement*	• Development/review of local IEC materials: emphasize benefits of newborn care services, mainly within the first week of life • Fee exemption for newborn care and functional referral system as well as for maternity care. • Strengthen community-based activities: education on hand washing and personal hygiene, behaviour change communication activities for educating caretakers on identification and prompt care seeking and to tackle harmful cultural beliefs, awareness campaigns using multiple channels to increase knowledge, demand for postnatal and community-based newborn care, and empower women. • Engage male partners in MNH care: Encourage male participation in ANC, labour, delivery and post natal visit.

### Leadership and governance bottlenecks and solutions

Policies for prescription and administration of antibiotics by qualified health workers only at referral (secondary or tertiary) hospitals, and not primary care level, were identified as one of the most important barriers to effective case management of neonatal infections by workshop participants (7 out of 12 country teams). Others included absence or poor dissemination (especially to primary care) of practice guidelines/protocols on treatment of neonatal infections.

Proposed solutions included development or review of national policies and guidelines on prevention and management of neonatal infections with the support of professional bodies. Country teams suggested these should include guidance on management of neonatal infections in primary health care facilities, including specification of the role of community health workers. In addition, workshop participants suggested disseminating guidelines to all health care providers, including in private settings.

### Health financing bottlenecks and solutions

Seven out of 10 country workshop participants reported insufficient funding for procurement, and continuous, sustainable distribution of antibiotics. High out-of-pocket expenses, arising from consultancy fees, transport, and treatment, as well as some unofficial payments, were a burden for families, especially for second and third line antibiotics. Limited coverage of financing schemes and insurance mechanisms for care of sick newborns was highlighted by all country teams.

Workshop participants called for more advocacy, engaging women's groups and professional associations, to support increased funding for newborn care and strengthen procurement and distribution of supplies and drugs, including antibiotics, at all levels of health care. Notably some countries with community-based insurance schemes, such as Indonesia, do not include newborns since registration takes a month.

A few country teams suggested that the challenge of out-of pocket expenses could be mitigated by including free consultation in medical colleges as part of a national treatment scheme, as is done in some countries such as Bangladesh and Vietnam, or establishing health insurance schemes including community-based insurance schemes.

### Health workforce bottlenecks and solutions

Eleven out of twelve country teams indicated a shortage of health care workers with adequate knowledge and skills to competently identify and manage newborn infections, especially in primary care settings. Retaining professional, well-trained health workers in rural areas is challenging. Mentorship and supervision structures were described as non-functional and incentives to motivate staff, support job satisfaction, and career development were lacking.

Workshop participants suggested that pre-service and in-service training are opportunities to reinforce knowledge and skills of health workers in newborn infection case management for primary, secondary and tertiary levels of care. Country teams suggested that well-trained, supervised and incentivised health workers could initiate oral antibiotic treatment in specific contexts where referral was not possible. Capacity strengthening and professional development of health care workers could be reinforced by improving supervision and mentoring, in collaboration with medical colleges and private institutions. In some settings, country teams suggested a computer-based training for ongoing in-service training and updates.

### Essential medical products and technologies bottlenecks and solutions

A common challenge reported was inefficiency of procurement and supply management systems (9 out of 12 country teams), leading to erratic antibiotic supply. There are manufacturing gaps, and limited distribution of supplies, as well as inadequate systems for forecasting and restocking. Consequently there are frequent stock-outs and prescription of alternative, second line antibiotics. There was also a need to strengthen laboratory services.

Proposed strategies include strengthening systems by improving management skills, warehousing capacity, and supporting a distribution system at subnational levels to ensure accurate forecasting and adequate management of stocks. Regular audits of medical stores and hospital pharmacies should be institutionalised, dependent on leadership and governance (discussed above).

### Health service delivery bottlenecks and solutions

A major concern from eight out of twelve country teams was inadequate referral systems between community, primary and secondary health facilities, causing delays in access to the appropriate level of care and treatment of newborn infections. There was inadequate recognition and referral of newborns with infection at primary health care facilities, partly due to lack of trained staff and supportive policy.

Quality of care was an important barrier for optimal management of neonatal infections at all levels of the health system. Country teams highlighted the absence of guidelines for neonatal infection management in health facilities and/or lack of adherence to these guidelines when available. Hospital-acquired infection prevention was not well implemented and audits of maternal-perinatal care were rare, and did not include the assessment for newborn infections based on risk factors.

Country teams suggested that increasing access to care should start with strengthened referral systems. Public-private sector partnerships and community-led transportation systems were suggested. Where referral is not possible, workshop participants suggested appropriately trained nurses and midwives may potentially be able to instigate treatment.

It was suggested that quality of care in facilities could be improved with regular use of checklists and audits of case management of neonatal infection, as well as perinatal death reviews. Guidelines for infection control, with adherence assessed through supervision by district health teams, were suggested.

### Health information system bottlenecks and solutions

There were ten out of twelve country teams who reported failure to collect data on management of neonatal infections, including data on antibiotic use, due to the lack of indicators for treatment of newborn infections in health information systems. Most country teams also highlighted that clinical records for sick and small newborns were inadequate at all levels of care.

Data collection and reporting tools should be reviewed to ensure indicators for treatment of newborn infection are part of routine systems. Electronic recording systems were suggested to strengthen data collection and reporting within facilities and to support evidence-based improvement of performance. Country teams proposed innovative tools, for example data collection via mobile phones by community health workers.

### Community ownership and partnership bottlenecks and solutions

Workshop participants from both Africa (3 countries) and South Asia (3 countries) highlighted the practice of a period of seclusion (usually around one month) after birth where mothers and babies stay indoors in some settings. If mothers or babies become ill during this period, care seeking is often delayed. This may be exacerbated by poor knowledge and recognition of signs of infections, delay in reaching appropriate care because of large distances to health facilities, and delay in receiving appropriate care and antibiotic treatment due to financial barriers (e.g. consultation fees in teaching hospitals or costs of second line antibiotics). Poor adherence to full treatment for neonatal infections was a challenge exacerbated by limited involvement of family members and communities.

Behaviour change activities should be strengthened, working with local community groups and leaders to emphasise the importance of early care-seeking for newborn illness and recognition of danger signs. Engagement of male partners during antenatal care, delivery, post discharge counselling, and postnatal visits is important. Inclusion of the benefits of early health care seeking for neonatal infection and adherence to treatment should be advocated by facility-based health care workers and communicated in local languages, empowering health workers at all levels.

## Discussion

To the best of our knowledge this is the first in-depth multi-country analysis of specific health system bottlenecks and strategies to address the management of neonatal infections. Neonatal infections are one of the main causes of neonatal deaths, and most of these deaths are in low-income countries where health systems are the most challenged. These twelve countries account for over 60% of the world's neonatal and maternal mortality and a similar proportion of stillbirths, and yet have varying contexts and health system strengths and weaknesses.

The participatory approach involved a wide range of programmatic and technical expertise. The process of the bottleneck analysis allowed for consensus-based data capture of context-specific challenges, barriers and possible solutions, from a diverse group of ground-level stakeholders and experts in twelve high burden countries. Together these countries account for the majority of maternal and newborn deaths [9]. Our results will sharpen health programme priorities and policies, and strengthen implementation of the *Every Newborn *Action Plan to end preventable neonatal deaths by 2035 [13], noting that many countries are already making health system changes [17].

These results suggest that community-level ownership and the health workforce are priority health system areas to improve to scale-up the treatment of neonatal infections. The lack of emphasis on newborn infection management at policy and programme level, lack of financial resources for care and quality data on neonatal infection case management, were also identified and graded by country teams as key challenges. It is essential that there is harmonisation and dissemination of newborn guidelines at all levels of care, and expansion of pre-service and in-service training for health care workers. Access to care must be improved, including by offering outpatient treatment for neonatal infection when referral is not possible, strengthening home-based newborn care, and engaging local communities to increase education and demand for quality healthcare services.

### Community ownership and partnership priority actions

It is important, even for hospital births (where women and their newborns may be discharged quickly), that women, families and communities are empowered to rapidly recognise and respond to danger signs if they occur. The time from first clinical signs to severe illness or death may be only a few hours. Maternal education, and engagement of fathers and extended family, will help families respond appropriately when their children are sick, including recognising danger signs with immediate care-seeking for treatment [18,19]. Prevention is beyond the scope of this paper, but community ownership and partnership have important roles in this too; for example hygiene and treatment of maternal infections and basic newborn care [15,22], which are described elsewhere in this series [24,25].

#### Referral systems

To achieve reductions in newborn mortality, strengthened referral systems are needed between all levels of care (primary, secondary and tertiary, see Figure 2), for rapid access to hospital based care for severe neonatal infections [26]. Barriers to adherence to referral include financial and logistical challenges as well as preference (with social and cultural influences). It is important to clearly define referral mechanisms (for example verbal, written, or referral slips), assess locally available resources for emergency communication (radio contact with facilities) and transport (community committees) to support implementation of community-based health programmes; including costs and logistics of transport and referral systems [20,23,27,28]. Several countries have shown that community-led referral systems or public-private partnerships can improve access to primary and secondary care. In Nigeria a loan fund was successfully linked with an initiative to involve local car owners in a stand-by transport scheme for emergency referrals [29]. In Mali, referral loan funds are managed by local health committees [30]. There is limited evidence on effectiveness, and implementation at scale of these referral mechanisms. The long-term objective should be to assess and establish operational referral systems for maternal, newborn and child emergencies (and elective referrals) as part of the district health system. Maximal benefits are achieved through improving both clinical care and referral systems [31,32].

### Health workforce priority actions

One of the greatest challenges is the gap in the availability and distribution of qualified health care workers to manage neonatal infections at all levels of health care (Figure 2). Management of neonatal infections, including administration of antibiotics, is usually restricted to doctors in secondary and tertiary hospitals. Our analysis showed that challenges to providing high quality care are often related to the low numbers of specialist staff with skills to manage newborn illness (midwives, trained nurses, paediatricians, neonatologists). The lack of trained nurses and a global neonatal nursing cadre is explored in greater detail elsewhere in the series, and is critical for supportive care such as safe administration of intravenous fluids and oxygen [33]. A strategy involving simple structural improvements, in-service training opportunities, effective team-based mentoring and supervision could strengthen the capacity of providers to care for newborns. The quality of primary health care is limited by weak supervision systems, poor implementation of guidelines and protocols and a lack of staff trained to diagnose and treat newborn infections. Trained community health workers and extension workers can play an important role in recognition of illnesses, supporting families to adopt healthy practices and seek care, encouraging delivery in a health care facility, identifying sick young infants and ensuring timely referral [27,34]. For example, in Nepal, neonates with PSBI were identified at home as needing treatment by well-trained facility-based workers and referred for care. Treatment was initiated in 90% of the PSBI episodes; and 93% of newborns completed a full course of gentamicin [35].

**Figure 5:**
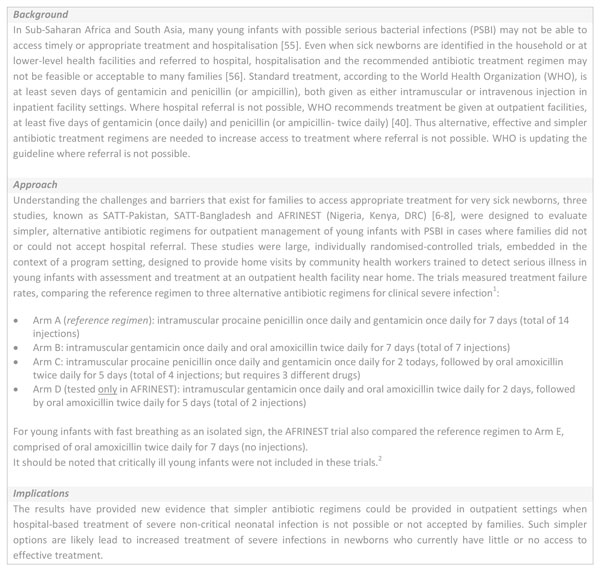
**Innovative treatment regimens for possible severe infections**. The Simplified Antibiotic Therapy Trial (SATT) and African Neonatal Sepsis Trial (AFRINEST) study groups. ^1^In a young infant (0-59 days old), at least one sign of severe infection (i.e. movement only when stimulated, not feeding well on observation, temperature greater than or equal to 38°C or less than 35.5°C or severe chest in-drawing. ^2^In a sick young infant, presence of any of the following signs - unconscious, convulsions, unable to feed at all, apnoea, unable to cry, cyanosis, bulging fontanelle, major congenital malformations inhibiting oral antibiotic intake, active bleeding requiring transfusion, surgical conditions needing hospital referral, persistent vomiting defined as vomiting following three attempts to feed the baby within 30 minutes. AFRINEST: African Neonatal Sepsis Trial; PSBI: possible serious infection; SATT: Simplified Antibiotic Therapy Trial; WHO: World Health Organization.

**Figure 6:**
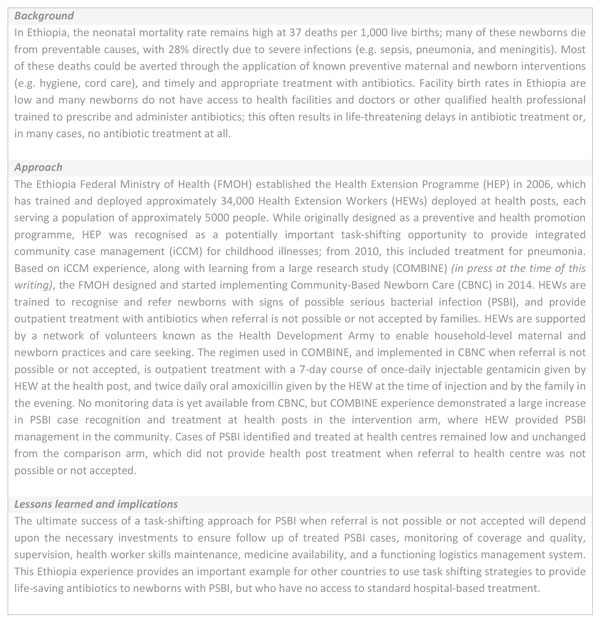
**Where referral is not possible: Task shifting the management of newborn potential serious infection to health extension workers in Ethiopia**. 1. Federal Ministry of Health: Health Extension program in Ethiopia. Health Extension and Education Center, Addis Ababa, Ethiopia June 2007 http://www.moh.gov.et/documents/36477/47416/HEW+profile+Final+08+07/da2fcc1a-6f38-4958-9ebf-a3310ec830c3;jsessionid = 054B89019E2E2ED40995C98D0D8D94B8?version = 1.0 2. Special Issue: Integrated Community Case Management (iCCM) at Scale in Ethiopia: Evidence and Experience. Ethiopia Medical Journal, Volume 52, Supplement 3, October, 2014. CBNC: community-based newborn care; FMOH: Federal Ministry of Health; HEP: health extension programme; HEWs: health extension workers; iCCM: integrated community case management; PSBI: possible serious bacterial infection.

There are some instances, however, when families may not, or cannot accept timely referral for care [6-8]. Management of neonates with PSBI with simpler antibiotic regimens where referral is not possible is a real need in many low resource settings. At present there is divergence across countries in policy and practice regarding outpatient treatment of neonatal infections; a recent review showed that 6 of 59 countries had permissive policies, and 11 of 59 countries (most in southern Asia) were implementing outpatient treatment of neonatal infections at different scales (sub-national or district level programmes) [36]. Recently large trials have reported that when hospital-based inpatient treatment is not feasible, simpler antibiotic regimens are effective when provided on an outpatient basis to non-critically sick neonates with PSBI, thereby increasing treatment for neonates who currently have little or no access to care (Figure 5) [6-8]. However, it is unlikely that one policy will meet the needs of all countries, given the different infrastructures, and a country-by-country approach is likely to be appropriate (Figure 6). Outpatient treatment should not become a substitute for hospital inpatient care (the standard of care), and requires close monitoring, supervision, and coordination with health facilities [34,37].

#### Guidelines and policies

Diagnosis, referral and treatment of neonatal infections is directed by international guidelines; primary-level recognition and management of danger signs have been added to integrated management of childhood illness (IMCI) guidelines [38]. IMCI has been shown to reduce neonatal mortality when implemented at high coverage [21,39,40], but lack of investment in recent years means it is not always fully implemented at peripheral level facilities, where it is most needed [41]. Guidelines in health facilities, and especially including job aids (e.g. gentamicin nomograms, or for intravenous fluid administration or nasogastric feeding) posted on the ward walls can help improve case management of sick neonates and children, and yet are often lacking, as noted in these 12 country assessments. Poor leadership support, staff shortages, and inadequate training and supervision to recognise and manage neonatal infections, as well as lack of basic supplies could be reasons for limited adherence to guidelines [42-44]. WHO is developing a guideline to treat non-critically sick neonates, where referral is not possible with simpler antibiotic regimens, which will soon be available.

At all levels of care there is a need for better management of drug supplies, with inclusion of neonatal formulations of antibiotics in facilities, and logistics monitoring and forecasting systems [45]. Safety concerns administering antibiotics are important and documentation, training and supervision are needed to ensure correct dosage and administration. Aminoglycoside antibiotics, such as gentamicin, are widely used and have the potential for toxicity above certain threshold levels, but data are limited [46] and routine therapeutic monitoring is often not done even in facilities in low and middle income countries. Improving availability of drugs with clear indications on neonatal dosage (especially gentamicin) may encourage greater adherence and simplify supply logistics [44].

### Other priority actions

Quality data are lacking on neonatal care, which limits the ability to track and evaluate service provision for the management of neonatal infections. Major data quality gaps include data on antibiotic use in the perinatal period and outcome data for neonatal infection. Demographic and Health Surveys (DHS) and Multiple Indicator Cluster Surveys (MICS) capture care seeking and treatment for fever and symptoms of acute respiratory infections for all children under five years, however, the sample is often too small for disaggregation, and data are not specifically presented for newborns. Improving availability of routine statistics through strengthening national/district health information systems should be a priority. More attention needs to be paid to the development and testing of indicators for treatment of neonatal infections and their inclusion in health management information systems to increase facility-based reporting [47]. Data collection through health workers using mobile phone technologies could improve quality data compilation and reporting at the health facility [48,49].

The lack of clinical audit and case review to assess the management of neonatal infection and the related deaths was highlighted by African country teams. The introduction of perinatal has the potential to reduce mortality when solutions are linked to action [50]. However, operationalising this in low-income countries remains a challenge [51], which is discussed in more detail elsewhere in the series[52].

### Limitations

The data generated from the workshop came from consensus views of participating national stakeholders including government representatives and experts and may be subjective. The quality and amount of information extracted from these workshops varied depending on the level of knowledge of participants on health system issues and facilitation. Sub-national data (two out of 28 states) received from India, the highest contributor of global neonatal deaths, were compiled as one input and may not be generalisable to the whole country; these two states are amongst the most marginalised and may represent a worst case scenario.

Another limitation was that bottlenecks were reported as perceived bottlenecks relative to other health system building blocks. There may be instances where known health system challenges, or deficits based on robust quantitative data, are in conflict with the perceived bottleneck grading. This could arise due to the method of grading relative to other health system building blocks, or because participants placed higher subjective value on other areas of their health system. Another explanation is that groups may view certain building block areas as easier to overcome, based on knowledge and experience of their setting, and the specific newborn intervention being discussed. The tool is comprehensive and detailed - which is one of its strengths. However, it also may have caused some workshop fatigue, particularly towards the end of the workshop where teams discussed and recorded solutions. For example, for the neonatal sepsis questionnaires, Pakistan completed the bottleneck portion of the questionnaires, but did not submit any solutions.

### Research agenda

The challenges and varying health system contexts discussed underline the need for implementation research on this topic, particularly around the "how to" questions. There is a need for better tracking of safety outcomes (for example after gentamicin use), monitoring of antimicrobial resistance and development of point of care diagnostics. WHO, UNICEF and Save the Children are facilitating several African and Asian countries to set up small scale demonstration sites for simplified management of sick newborns with PSBI as outpatients, where referral is not possible. Coverage data for treatment of newborn infections are lacking and it may not be feasible to use household surveys [53]; improving the metrics for tracking programmatic quality is crucial [47].

## Conclusions

**Figure 7:**
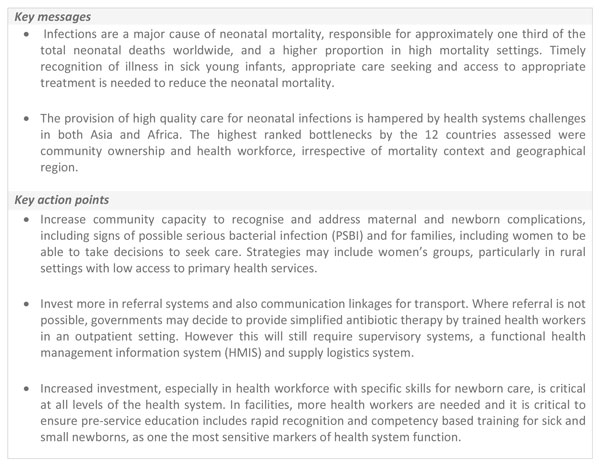
**Key messages and action points for treatment of neonatal infections**.

To reduce the burden of neonatal mortality, improving management, as well as preventing, neonatal infections is essential. Key messages and action points from this paper are summarised in Figure 7. Even with a weak health system, measurable mortality reduction can be achieved by starting with communities and primary care, up to tertiary levels, and addressing the highest priority health system bottlenecks; a skilled workforce and community participation and ownership. There is a critical need for improved access to antibiotic treatment at all levels of care, in addition to supportive care, with strong referral systems. There is an urgent need to increase investment, and to train more health care workers in neonatal care. Every country must prioritise assessment of their health system and ensure a programme is being implemented to reach every newborn.

## List of abbreviations

DHS: Demographic and Health Surveys; DRC: Democratic Republic of Congo; HEWs: Health Extension Workers; IMCI: Integrated Management of Childhood Illness; MICS: Multiple Indicator Cluster Surveys; NMR: Neonatal Mortality Rate; PSBI: possible serious bacterial infection; WHO: World Health Organization.

## Competing interests

All authors declare they have no competing interests. The assessment of bottlenecks expressed during consultations reflects the perception of the technical experts and may not be national policy. The authors alone are responsible for the views expressed in this article and they do not necessarily represent the decisions, policy or views of the organisations listed, including WHO.

## Authors' contributions

AS-K was responsible for the tool development, conceptualisation of the paper, substantial contributions to the data analysis, and writing. ACS oversaw the analysis and writing of the paper; CN contributed to data process, analysis, and literature review; KED was responsible for the overall coordination of the country consultation process, bottleneck analysis tool development and reviews of the paper drafts. SGM, SW, GLD, SQ, MY, JEL contributed sections of text and reviewed paper drafts. All named authors contributed to paper drafts and approved the final manuscript.

## Supplementary Material

Additional file 1 Bottleneck tool questionnaire Click here for file

Additional file 2 Supplementary tables and figures. Click here for file

## Declarations

Publication of this supplement was funded by the Bill and Melinda Gates Foundation through a grant to US Fund for UNICEF (Grant ID: OPP1094117), and support from Save the Children's Saving Newborn Lives Programme. Additional funding for the bottleneck analysis was received from USAID (Grant ID: GHA-G-00-07-00007) through UNICEF.

This article has been published as part of *BMC Pregnancy and Childbirth *Volume 15 Supplement 2, 2015: Every Woman, Every Newborn. The full contents of the supplement are available online at http://www.biomedcentral.com/bmcpregnancychildbirth/supplements/15/S2.
