# Supplementary material for: Treatment of neonatal infections: a multi-country analysis of health system bottlenecks and potential solutions
Source: BMC Pregnancy Childbirth. 2015 Sep 11;15(Suppl 2):S6. doi: 10.1186/1471-2393-15-S2-S6 (PMC4578441; doi:10.1186/1471-2393-15-S2-S6)
Supplement: Additional file 2 — Supplementary tables and figures. [file 1471-2393-15-S2-S6-S2.docx]

Treatment of neonatal infections: a multi-country analysis of health system bottlenecks and solutions

Additional file 2

A. Table S1: Bottlenecks for treatment of neonatal infections 2

B. Table S2: Significant bottlenecks and solution themes by health systems building blocks 12

C. Table S3: Health system areas of challenges highlighted by countries 14

D. Table S4: Solutions for treatment of neonatal infections 17

E. Figure S1: Subnational grading of bottlenecks for treatment of neonatal infections 19

F. Table S5: Evidence for solutions for treatment of neonatal infections 20

# A. Table S1: Bottlenecks for treatment of neonatal infections

| Healthy System Building Block | Category | Bottlenecks | Africa | | | | | |  | Asia | | | | | |
| --- | --- | --- | --- | --- | --- | --- | --- | --- | --- | --- | --- | --- | --- | --- | --- |
|  |  |  | Cameroon | DRC | Kenya | Malawi | Nigeria | Uganda | Afghanistan | | Bangladesh | India | Nepal | Pakistan | Vietnam |
| Leadership and Governance  Leadership and Governance  Leadership and Governance | **Policy/strategy** | Lack of policy for management of neonatal infections and other supportive policies like discharge policy |  |  |  |  |  |  |  | |  | A |  | A, G, K, P, S |  |
|  |  | Policy stipulation that newborns with severe infections are currently referred and managed as in-patients in secondary and tertiary health facilities |  |  |  |  |  |  |  | |  |  |  |  |  |
|  |  | In the RMNCH strategy, the specific identification of major causes of severe infection are not well reflected |  |  |  |  |  |  |  | |  |  |  |  |  |
|  | **Guidelines and dissemination** | No national guidelines by level of care |  |  |  |  |  |  |  | |  |  |  | G, K, P |  |
|  |  | Lack of integration of guidelines in private health facilities |  |  |  |  |  |  |  | |  |  |  |  |  |
|  |  | Poor/limited dissemination of national guidelines (e.g. does not reach providers at lower levels) |  |  |  |  |  |  |  | |  | A |  |  |  |
|  |  | Following guidelines varies from institution to institution due to technical limitations (e.g. discharge timing, antibiotic use) |  |  |  |  |  |  |  | |  | O |  |  |  |
|  | **Protocol** | No national harmonised operation protocol for severe newborn infections |  |  |  |  |  |  |  | |  |  |  | G |  |
|  |  | No hospital protocol including procedures in referral / referrals not as per guidelines |  |  |  |  |  |  |  | |  | O |  |  |  |
|  |  | Out-dated clinical protocols |  |  |  |  |  |  |  | |  |  |  |  |  |
|  | **Policy restrictions (prescription)** | Prescription restrictions for antibiotics (only physicians authorised, CHWs not able to prescribe/use) |  |  |  |  |  |  |  | |  | A |  | B |  |
|  | **Lack of care at particular levels** | Infection prevention in hospitals not well addressed |  |  |  |  |  |  |  | |  |  |  |  |  |
|  |  | Sepsis is not managed at health centre level even though midwives and medical assistants may be able to treat |  |  |  |  |  |  |  | |  |  |  |  |  |
|  |  | Pre-referral treatment not yet available at community level |  |  |  |  |  |  |  | |  |  |  |  |  |
|  |  | Home-based postnatal visits not being carried out by CHWs |  |  |  |  |  |  |  | |  |  |  |  |  |
|  | **Awareness** | Lack of awareness |  |  |  |  |  |  |  | |  |  |  | S |  |
|  | **Private sector** | Private sector is not involved |  |  |  |  |  |  |  | |  |  |  |  |  |
|  |  | Lack of integration of guidelines in private health facilities |  |  |  |  |  |  |  | |  |  |  |  |  |
|  | **Infrastructure** | Inadequate infrastructure for care of babies with severe infections |  |  |  |  |  |  |  | |  |  |  | S |  |
| Health Financing  Health Financing | **Funding** | Insufficient/lack of funds for procurement and distribution of drugs |  |  |  |  |  |  |  | |  |  |  | G |  |
|  |  | Insufficient allocation/ disbursement of funds (e.g. for training staff at all levels) |  |  |  |  |  |  |  | |  | A |  | G |  |
|  |  | Find flow from national to state level does not commensurate with fund allocation and time frame |  |  |  |  |  |  |  | |  | O |  |  |  |
|  |  | Absence of subsidies / lack of free treatment / scaled up schemes of severe infections at all levels of the government |  |  |  |  |  |  |  | |  |  |  | S |  |
|  |  | Lack of financial resources of caregivers |  |  |  |  |  |  |  | |  |  |  |  |  |
|  |  | No specific funding for lab support and blood component availability |  |  |  |  |  |  |  | |  | O |  |  |  |
|  | **Costs/ fees** | High cost of medications |  |  |  |  |  |  |  | |  |  |  | B, G |  |
|  |  | High cost of transport |  |  |  |  |  |  |  | |  |  |  |  |  |
|  |  | High cost of testing and services |  |  |  |  |  |  |  | |  |  |  |  |  |
|  | **Out-of-pocket expenses / user fees** | High out-of-pocket expenses / burden on families when medicines are not available |  |  |  |  |  |  |  | |  |  |  | G, K, P |  |
|  |  | User fees represent barrier to admission of sick newborns (e.g. at medical colleges) |  |  |  |  |  |  |  | |  | O |  |  |  |
|  | **Awareness** | Clients and service providers not aware of free entitlements and available funds |  |  |  |  |  |  |  | |  | A |  |  |  |
| Health Workforce  Health Workforce | **Number, distribution and role of health workers** | Shortage of competently trained / skilled health workers to manage severe infections in newborns (lower level facilities, poorer competence, lack of skills, ban on recruitment of new posts) |  |  |  |  |  |  |  | |  |  |  | A, K, S |  |
|  |  | Poor distribution/ posting of personnel (urban vs. rural, not enough staff in community) |  |  |  |  |  |  |  | |  | A |  | B |  |
|  |  | Lack in clarity of roles of health worker |  |  |  |  |  |  |  | |  |  |  |  |  |
|  | **Mentoring and supervision** | Mentoring and supervision guidelines incomplete / not in place |  |  |  |  |  |  |  | |  | O |  |  |  |
|  |  | Supervision for mentoring protocols not institutionalised |  |  |  |  |  |  |  | |  |  |  |  |  |
|  |  | Weak/lack of supervision and mentorship (supervision not specific to infections) |  |  |  |  |  |  |  | |  | A |  | B, K, S |  |
|  | **Accountability** | No accountability / not following protocols |  |  |  |  |  |  |  | |  |  |  | G, S |  |
|  | **Incentives and motivation** | Poor health worker attitude and motivation |  |  |  |  |  |  |  | |  |  |  | P |  |
|  |  | Task shifting |  |  |  |  |  |  |  | |  |  |  |  |  |
|  |  | Lack of compensation and incentives |  |  |  |  |  |  |  | |  | O |  |  |  |
|  | **Training** | No standard/uniform training and refresher training (particularly at community/lower levels) |  |  |  |  |  |  |  | |  | A,O |  | A, G, P, S |  |
|  | **Guidelines and instructions** | Lack of guidelines, instructions, job charts and job aids for newborn infection management |  |  |  |  |  |  |  | |  | O |  |  |  |
|  |  | Lack of job descriptions or not known to all |  |  |  |  |  |  |  | |  | A |  | K |  |
|  |  | No system to promote adherence to standard treatment guidelines |  |  |  |  |  |  |  | |  |  |  | G |  |
| Essential Medical Products and Technologies  Essential Medical Products and Technologies | **Lack of supplies and equipment** | Stock-outs / irregular supply of medicines/antibiotics |  |  |  |  |  |  |  | |  | A, O |  | A, G, K, P |  |
|  |  | Antibiotics not available at all levels |  |  |  |  |  |  |  | |  |  |  |  |  |
|  |  | Electronic weighing machine not available in all units |  |  |  |  |  |  |  | |  | A |  |  |  |
|  |  | Lack of beds |  |  |  |  |  |  |  | |  |  |  |  |  |
|  | **Logistical management** | Lack of system for forecasting and procurement of essential commodities (including check and balance and tracking) |  |  |  |  |  |  |  | |  | O |  | B, S |  |
|  |  | Issues with supply chain management |  |  |  |  |  |  |  | |  | A |  |  |  |
|  |  | Lack of effective system for distribution |  |  |  |  |  |  |  | |  |  |  | S |  |
|  |  | Overuse of first line treatment – not sustainable or continuous |  |  |  |  |  |  |  | |  |  |  | A |  |
|  | **NEML restrictions** | NEML recommends use of injectable antibiotics in secondary and tertiary hospitals only |  |  |  |  |  |  |  | |  |  |  |  |  |
| Health Service Delivery  Health Service Delivery | **Staff capacity** | Insufficient capacity of staff to carry out assessments and care |  |  |  |  |  |  |  | |  |  |  | S |  |
|  | **Referrals, postnatal check-ups and home visits** | Problems with referral system – delays due to caretakers not being aware of danger signs and lack of transport |  |  |  |  |  |  |  | |  | O |  | K |  |
|  |  | Delayed/missed postnatal check-ups |  |  |  |  |  |  |  | |  |  |  |  |  |
|  |  | Pre-referral treatment not available in village clinics |  |  |  |  |  |  |  | |  |  |  |  |  |
|  |  | Lack of home visits that prevent early detection of neonatal infections |  |  |  |  |  |  |  | |  |  |  |  |  |
|  | **Protocols / standards** | Lack of dissemination and utilisation of protocols/standards |  |  |  |  |  |  |  | |  |  |  |  |  |
|  |  | Lack of monitoring system to promote and monitor adherence to protocols |  |  |  |  |  |  |  | |  |  |  | S |  |
|  |  | Protocol for administration of specific antibiotics is not clear |  |  |  |  |  |  |  | |  |  |  |  |  |
|  | **Quality** | Quality services are not available at particular facilities as per standards |  |  |  |  |  |  |  | |  | A |  |  |  |
|  |  | Quality of care checklists require regular review and upgrading |  |  |  |  |  |  |  | |  |  |  |  |  |
|  | **Health worker attitudes** | Health worker attitudes |  |  |  |  |  |  |  | |  | A, O |  | B |  |
|  |  | Ineffective communication (with clients and between community and facility) |  |  |  |  |  |  |  | |  | A |  |  |  |
|  | **Access** | Scattered community |  |  |  |  |  |  |  | |  |  |  |  |  |
|  | **Follow-up** | No follow-up |  |  |  |  |  |  |  | |  |  |  | G |  |
| Health Information System  Health Information System | **HMIS** | Does not capture neonatal infection indicators/information |  |  |  |  |  |  |  | |  |  |  | A, S |  |
|  | **Records** | Not included in records / records not maintained |  |  |  |  |  |  |  | |  | A |  | A, B, P |  |
|  | **Audits / critical review** | Not included in audits |  |  |  |  |  |  |  | |  | O |  | B, P |  |
|  |  | Lack of critical review of appropriate management of severe newborn infections |  |  |  |  |  |  |  | |  | A, O |  |  |  |
|  | **Sources / repositories of information** | No neonatal register / repository of health information |  |  |  |  |  |  |  | |  |  |  | K |  |
|  |  | No checklists or SOPs |  |  |  |  |  |  |  | |  |  |  | G, S |  |
|  |  | Partners/ community-based organisations have data not reported to MoH |  |  |  |  |  |  |  | |  |  |  |  |  |
|  | **Reporting** | Weak coordination between different agencies in reporting of information |  |  |  |  |  |  |  | |  |  |  |  |  |
|  | **Quality** | Poor quality of data and lack of verification |  |  |  |  |  |  |  | |  |  |  | S |  |
|  | **Public private partnership (PPP)** | PPP not available for newborn care |  |  |  |  |  |  |  | |  | O |  |  |  |
| Community Ownership and Partnership  Community Ownership and Partnership  Community Ownership and Partnership | **No/poor promotion** | No IEC in local language |  |  |  |  |  |  |  | |  |  |  |  |  |
|  |  | Lack of community mobilisation |  |  |  |  |  |  |  | |  | O |  | S |  |
|  | **Financial barriers** | Community programs limited to a few districts due to financial constraints |  |  |  |  |  |  |  | |  |  |  | G |  |
|  | **Awareness** | Lack of awareness |  |  |  |  |  |  |  | |  |  |  | A |  |
|  |  | Inadequate efforts to improve community awareness for the importance of postnatal care |  |  |  |  |  |  |  | |  |  |  | B |  |
|  | **Acceptability** | Lack of comprehensive info on acceptability |  |  |  |  |  |  |  | |  |  |  |  |  |
|  |  | Not identified as an acceptable intervention in community |  |  |  |  |  |  |  | |  |  |  |  |  |
|  | **Referral and transport** | Poor referral and transport system preventing timely treatment (including long distance to facility, poor linkages between community and facility) |  |  |  |  |  |  |  | |  |  |  | K |  |
|  | **Poor compliance / health seeking** | Poor health seeking / delay in decision to go to facility / poor compliance in receiving full treatment |  |  |  |  |  |  |  | |  | A |  |  |  |
|  | **Training** | No training a community level to identify severe infection |  |  |  |  |  |  |  | |  |  |  | G |  |
|  | **Coordination** | Lack of strategies and support to facilitate the use of services, particularly by newborns living in rural / remote areas |  |  |  |  |  |  |  | |  |  |  |  |  |
|  | **Socio-cultural barriers and perceptions** | Socio-cultural barriers (postnatal period not perceived as priority after vaginal delivery, misconceptions, seclusion of newborns, challenges faced by women, etc) |  |  |  |  |  |  |  | |  | A, O |  |  |  |
|  | **Access** | Inability to access services at all levels |  |  |  |  |  |  |  | |  |  |  |  |  |
|  | **Participation/ involvement** | Poor involvement of men and community |  |  |  |  |  |  |  | |  | A |  | P |  |

# B. Table S2: Significant bottlenecks and solution themes by health systems building blocks

| **Health system Building block** | **Subcategories of bottlenecks** | **Number of countries** | **Solutions themes** |
| --- | --- | --- | --- |
| **Leadership and governance** | - Outdated/lack of dissemination of Clinical guidelines/ protocols - Policy restrictions for prescription/ administration of antibiotics | 7 | *Policy/guidelines review, harmonization and dissemination* |
|  |  | 5 | *Task shifting policy* |
| **Health finance** | - Inadequate flow/ insufficient allocation of funds | 7 | *Funding for newborn health* |
|  | - Out-of pocket expenditures | 9 | *National insurance schemes expansion* |
| **Health workforce** | - Poor distribution and limited availability of skilled health workers (nurses and pediatricians) | 11 | *Local recruitment of health workers* |
|  | - Lack of /poor knowledge of existing guidelines in health facilities | 4 | *Quality assurance/ improvement* |
|  | - Lack of motivation and incentives | 5 | *Institutionalization of incentives* |
|  | - Insufficient competency-based training; weak supervision and mentoring mechanisms | 9 | *Competency-based skilled based pre-in service training* |
| **Essential medical products and technologies** | - Frequent stock-outs of antibiotics | 11 | *Quality control mechanisms*  *Strengthening procurement and supply chain management system* |
|  | - Ineffective forecasting and procurement system at all levels of care | 8 |  |
|  | - Inadequate storage infrastructures and distribution mechanisms | 5 |  |
| **Health service delivery** | - Lack of infrastructure for care of sick newborns / short hospital stay after delivery | 5 | *Mobile outreach MNH services/ post-discharge counselling* |
|  | - Ineffective referral system from community to secondary facilities | 8 | *Two way referral system for sick newborns* |
|  | - Community-based support for sepsis case management not defined or adequately integrated in health clinics | 6 | *Scale-up community/home-based maternal and newborn care* |
|  | - Low quality of care (no checklist, lack of SOPs, slow adherence to guidelines) | 5 | *Quality assurance/ improvement mechanisms* |
| **Health management information system** | - No defined indicator in HMIS or lack of repository for data on neonatal sepsis | 10 | *Develop electronic reporting systems* |
|  | - Lack of review process; sepsis case management not integrated in perinatal audits | 7 | *Review processes and evidence-based decision -making* |
| **Community ownership and participation** | - Lack of awareness and knowledge on danger signs for neonatal infections | 6 | *Development/review of local IEC materials* |
|  | - Poor access to /use of newborn care services | 7 | *Fee exemption for newborn care and functional referral system* |
|  | - Limited engagement of male partners | 7 | *Male participation and involvement* |
|  | - Socio-cultural factors | 6 | *Community-based education for behaviour change* |
|  | - Poor compliance in receiving full treatment | 4 | *Education and sensitization* |

# C. Table S3: Health system areas of challenges highlighted by countries

| **Health system building blocks** | **Sub-category** | Africa | | | | | | Asia | | | | | |
| --- | --- | --- | --- | --- | --- | --- | --- | --- | --- | --- | --- | --- | --- |
|  |  | **Cameroon** | **DRC** | **Kenya** | **Malawi** | **Nigeria** | **Uganda** | **Afghanistan** | **Bangladesh** | **India** | **Nepal** | **Pakistan** | **Vietnam** |
| **Leadership and governance** | Policy development/ revision |  |  |  |  |  |  |  |  |  |  |  |  |
|  | Policy dissemination/ implementation |  |  |  |  |  |  |  |  |  |  |  |  |
|  | Policy restriction |  |  |  |  |  |  |  |  |  |  |  |  |
|  | Awareness/advocacy |  |  |  |  |  |  |  |  |  |  |  |  |
|  | Private sector engagement |  |  |  |  |  |  |  |  |  |  |  |  |
| **Health finance** | Funding availability / Allocation |  |  |  |  |  |  |  |  |  |  |  |  |
|  | Out-of pocket expenditures |  |  |  |  |  |  |  |  |  |  |  |  |
|  | Coverage of financing schemes |  |  |  |  |  |  |  |  |  |  |  |  |
| **Health workforce** | Availability |  |  |  |  |  |  |  |  |  |  |  |  |
|  | Distribution |  |  |  |  |  |  |  |  |  |  |  |  |
|  | Job aids/charts |  |  |  |  |  |  |  |  |  |  |  |  |
|  | Skills and competency |  |  |  |  |  |  |  |  |  |  |  |  |
|  | Motivation/incentives |  |  |  |  |  |  |  |  |  |  |  |  |
|  | Supervision and mentoring - BOTH |  |  |  |  |  |  |  |  |  |  |  |  |
|  | Role of community health workers |  |  |  |  |  |  |  |  |  |  |  |  |
| **Essential medical products and technologies** | National Essential  Medical List |  |  |  |  |  |  |  |  |  |  |  |  |
|  | Availability |  |  |  |  |  |  |  |  |  |  |  |  |
|  | Supply chain |  |  |  |  |  |  |  |  |  |  |  |  |
|  | Costs of drugs |  |  |  |  |  |  |  |  |  |  |  |  |
| **Health service delivery** | Availability of services (including space & equipment) |  |  |  |  |  |  |  |  |  |  |  |  |
|  | Referral |  |  |  |  |  |  |  |  |  |  |  |  |
|  | Outreach/community |  |  |  |  |  |  |  |  |  |  |  |  |
|  | Quality of care |  |  |  |  |  |  |  |  |  |  |  |  |
| **Health management information system** | Indicator |  |  |  |  |  |  |  |  |  |  |  |  |
|  | Registers |  |  |  |  |  |  |  |  |  |  |  |  |
|  | Completeness (private sector and community) |  |  |  |  |  |  |  |  |  |  |  |  |
|  | Review process |  |  |  |  |  |  |  |  |  |  |  |  |
| **Community ownership and participation** | Awareness and knowledge |  |  |  |  |  |  |  |  |  |  |  |  |
|  | Community engagement |  |  |  |  |  |  |  |  |  |  |  |  |
|  | Barriers to access /use care |  |  |  |  |  |  |  |  |  |  |  |  |
|  | Socio-cultural factors |  |  |  |  |  |  |  |  |  |  |  |  |
|  | Perception |  |  |  |  |  |  |  |  |  |  |  |  |
|  | Care seeking |  |  |  |  |  |  |  |  |  |  |  |  |
|  | Compliance |  |  |  |  |  |  |  |  |  |  |  |  |

# D. Table S4: Solutions for treatment of neonatal infections

| **Health system building blocks** | **Solutions proposed** |
| --- | --- |
| **Leadership and governance** | - **Review / formulation of policies and strategies:** integration of neonatal care into integrated management of childhood illnesses (IMCI) programs; expansion/implementation of community-based MNH programs (NGA, PAK, CAM) - **Harmonize guidelines for management of neonatal infections at all level of care:** engage professional bodies and private health care institutions (COD, IND)      - **Ensure effective dissemination of guidelines:** dissemination through workshops in collaboration with district health management teams, websites with regular updates (IND, KEN, C0D) |
| **Health financing** | - **Increase budget allocated for newborn health**: ensure adequate resources for trainings of health workers, laboratory services, and commodities including injectable antibiotics (BGD, COD, KEN, ND, PAK, VTN) - **Expand health insurance schemes to address out-of-pocket payments**: expansion of community-based health insurance schemes (NGA, COD); inclusion of medical college fees and laboratory tests into insurance schemes (IND, NGA, VTN) |
| **Health workforce** | - **Recruit local staff residing in the community** to expand the availability of health care workers in all areas (IND) - **Develop an electronic human resources training database** linked to the profile of health care workers to ensure all staff are trained (IND) - **Conduct large scale pre-service and in-service trainings of health care workers in newborn care** including management of neonatal infections; Enhance the quality of trainings provided through supervision, mentoring and certification systems; involve private medical colleges and institutions; Train community-based health care workers in newborn care (BGD, NGA, KEN, VTN, IND, DRC, CAM) - **Motivate of health providers through institutionalization of incentives** to improve their retention in rural and hard to reach areas such as improved welfare packages or wages, hardship allowances, pay for performance (NGA, IND, KEN) |
| **Health service delivery** | - **Ensure post-discharge counselling on danger signs** for newborns (IND); - **Scale-up newborn care at the lower level of care**: Strengthen the provision of newborn care, especially sepsis case management at primary health care level, institutionalize home visits / domiciliary care by trained personnel; Expand mobile outreach MNH services to make newborn services closer to the community (KEN, NGA, PAK, CAM, COD) - **Strengthen two way referral system** for sick newborns (IND, KEN, VTN, COD, NGA); - **Establish quality assurance mechanisms**: a quality assurance cell at the state/national level with regular quality assessments with emphasis on supportive supervision and mentoring by medical colleges and private hospitals (IND); national scale-up of clinical audits and perinatal death reviews (BGD, CAM); Periodic critical review of appropriate management of newborn infections (NGA); Regular review and dissemination of quality of care check lists (NGA); |
| **Essential medical products and technologies** | - **Strengthen the national procurement and supply system**: Forecast adequate amount of injectable antibiotics according to the needs for treatment and based on the buffer to be kept (NGA, VTN, IND); Streamline the procurement process including fast tracking of essential commodities (KEN, COD, NGA, CAM); Special equipment like, CPAP, portable x-ray, USG and arterial blood gas analyser should made available for tertiary care centres (IND); - **Establish quality control mechanisms**: Auditing of medical stores; facility assessments of those reporting frequent stock-outs; assessment of the quality of antibiotics in the bidding process; ensure the delivery of quality products (VTN, IND, PAK); Develop an electronic logistic management system (NGA, IND); |
| **Health management information system** | - **Strengthen data collection and reporting for newborn care:** disaggregate the data in the health management information system to include newborn health interventions, especially management of severe neonatal infections; add community data and postnatal consultations; develop neonatal registers; and set up a monitoring system for hospital infection prevention (COD, KEN, CAM, VTN, PAK) - **Develop electronic reporting system:** software apps for record keeping on newborn interventions including neonatal sepsis management and establishing linkages from facility to community (IND) - **Establish review processes; analyse and use data on management of neonatal infections** to inform performance review meetings and for quality improvement processes (PAK) - **Enhance research in newborn care:** conduct bacterial surveillance and antibiotics resistance studies; conduct newborn survival analysis to inform program managers (NGA, VTN) |
| **Community ownership and participation** | - **Development/review of local IEC materials**: emphasize benefits of newborn care services, mainly within the first week of life (NGA) - Fee exemption for newborn care and functional referral system (address under finance and service delivery - **Strengthen community-based activities**: education on hand washing and personal hygiene, behaviour change communication activities for educating caretakers on identification and prompt care seeking and to tackle harmful cultural beliefs, awareness campaigns using multiple channels to increase knowledge, demand for postnatal and community-based newborn care, and empower women (BGD, NGA, VTN, PAK, KEN) - **Engage male partners in MNH care**: Encourage male participation in ANC, labour, delivery and post natal visit (IND, NGA, COD, CAM) |

# E. Figure S1: Subnational grading of bottlenecks for treatment of neonatal infections


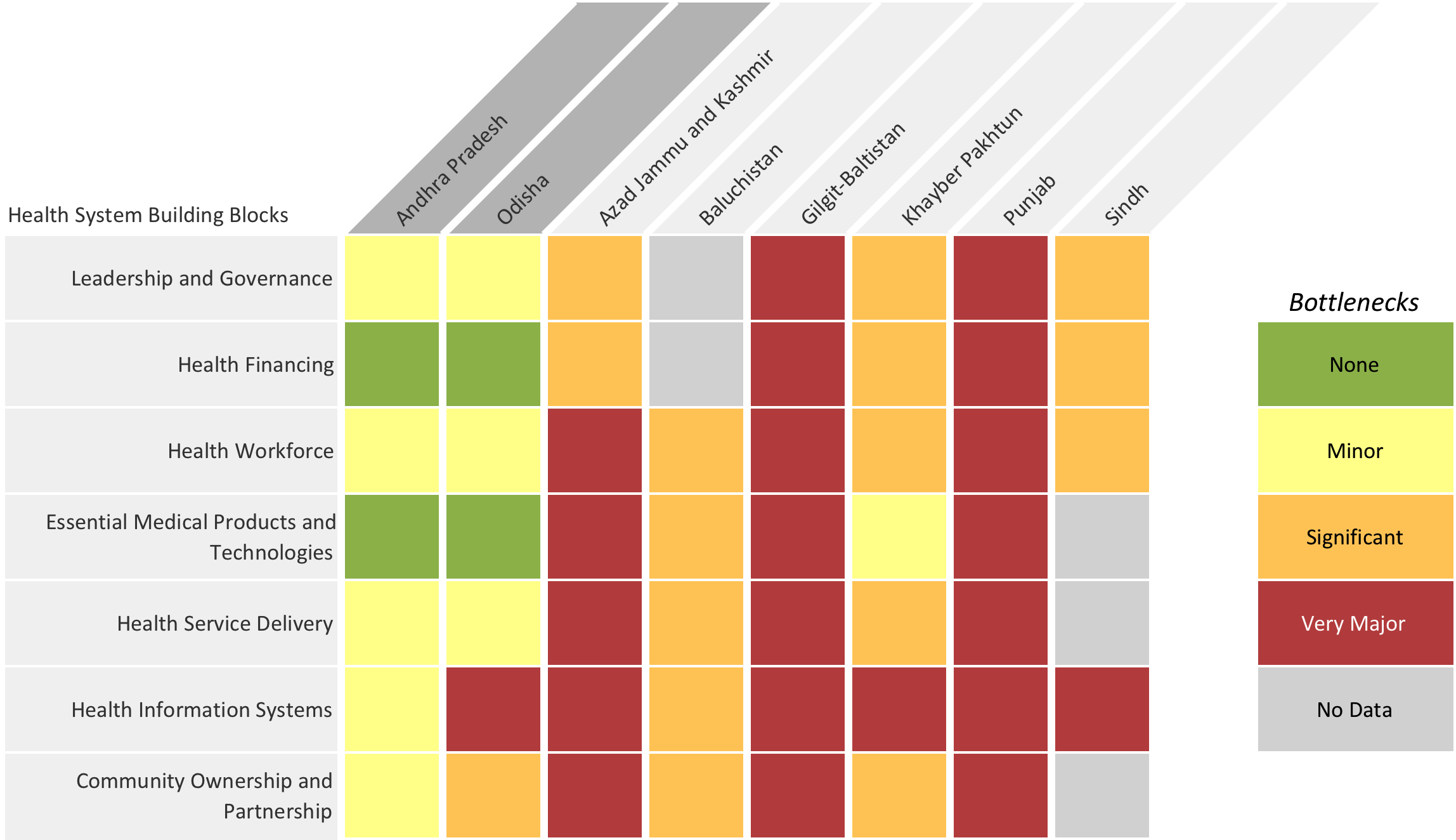


# F. Table S5: Evidence for solutions for treatment of neonatal infections

| **Health system Building blocks** | **Sub-category** | **Solutions** | **Evidence for solutions** | **Source** |
| --- | --- | --- | --- | --- |
|  |  |  |  |  |
|  |  |  |  |  |
|  |  |  |  |  |
| **Leadership and governance** | Lack of /outdated clinical protocols for management of neonatal infections | ***Review / formulation of policies and strategies:*** *integration of neonatal care into integrated management of childhood illnesses (IMCI) programs; expansion/implementation of community-based MNH programs (NGA, PAK, CAM)* | - 36% of expected newborns less than 2 months old sought care in 59 HFs in Sarlahi compared to 14% in other CB-IMCI districts which do not have the newborn care package. | [Johnsnowinc (http://www.jsi.com/JSIInternet/Inc/Common/_download_pub.cfm?id=12143&lid=3)](http://www.jsi.com/JSIInternet/Inc/Common/_download_pub.cfm?id=12143&lid=3) |
|  |  |  | - Of the 499 cases less than 2 months old who presented at HFs, 29% were classified as possible severe bacterial infection (PSBI), of which 87% were either treated at peripheral HFs or referred to a higher level. |  |
|  |  |  | - On average 1.7 newborn cases (n=64) were referred by the FCHVs interviewed during this 9 month period. | Knippenberg, Rudolf, et al. "Systematic scaling up of neonatal care in countries." *The Lancet* 365.9464 (2005): 1087-1098. (To look at) |
|  |  |  | - Of the sick newborns brought to the FCHVs, 76% were brought within 3 days of onset of illness. |  |
|  |  |  | - Of the newborn cases that the FCHVs referred to the HF (n=39), caretakers took 54% to a MOHP HF, 44% to a private clinic, and 3% to the hospital. All caretakers reported seeking further care for their sick newborns based on the advice given by the FCHVs. |  |
|  |  |  | - Community health workers were trained to conduct postnatal home visits and women’s group meetings; physicians, nurses, and community health workers were trained to treat or refer sick newborns and children; supply of drugs and supervision were strengthened. The infant mortality rate (adjusted hazard ratio 0.85, 95% confidence interval 0.77 to 0.94) and the neonatal mortality rate beyond the first 24 hours (adjusted hazard ratio 0.86, 0.79 to 0.95) were significantly lower in the intervention clusters than in control clusters. The adjusted hazard ratio for neonatal mortality rate was 0.91 (0.80 to 1.03). A significant interaction was found between the place of birth and the effect of the intervention for all mortality outcomes except post-neonatal mortality rate. The neonatal mortality rate was significantly lower in the intervention clusters in the subgroup born at home (adjusted hazard ratio 0.80, 0.68 to 0.93) but not in the subgroup born in a health facility (1.06, 0.91 to1.23) (P value for interaction=0.001). Optimal newborn care practices were significantly more common in the intervention clusters. | Bhandari, Nita, et al. "Effect of implementation of Integrated Management of Neonatal and Childhood Illness (IMNCI) programme on neonatal and infant mortality: cluster randomised controlled trial." BMJ 344 (2012). |
|  | Limited dissemination / implementation of guidelines with low private sector engagement | ***Harmonise guidelines for management of neonatal infections at all level of care:*** *engage professional bodies and private health care institutions (COD, IND)* |  |  |
|  |  |  |  |  |
|  |  | ***Ensure effective dissemination of guidelines:*** *dissemination through workshops in collaboration with district health management teams, websites with regular updates (IND, KEN, C0D)* |  |  |
|  | Policy restrictions for prescription/ administration of antibiotics |  |  |  |
| **Health finance** | Insufficient allocation of funds for procurement of drugs and laboratory services/Inadequate funds flow to the lower level | ***Increase budget allocated for newborn health****: ensure adequate resources for trainings of health workers, laboratory services, and commodities including injectable antibiotics (BGD, COD, KEN, ND, PAK, VTN)* |  |  |
|  | High out-of pocket expenditures for consultancy fees and high cost of antibiotics (2^nd^ & 3^rd^ lines) | ***Expand health insurance schemes to address out-of-pocket payments****: expansion of community-based health insurance schemes (NGA, COD); inclusion of medical college fees and laboratory tests into insurance schemes (IND, NGA, VTN)* | - In Senegal, approximately 93% of insurance scheme members with delivery coverage delivered at a modern health facility versus 71% of non-members and members without delivery coverage. In Mali, 94% of members and 65% of non-members had a facility delivery. In Ghana, a higher proportion of members (75%) than non-members (65%) reported delivering at a modern health facility, but this difference was not statistically significant. Interestingly, in Ghana, while 91% of women reported having a normal delivery, members of the Nkoranza insurance scheme were twice as likely as Nkoranza uninsured women to have a caesarean delivery, and five times more likely than women in Offinso district. | Smith, Kimberly V., and Sara Sulzbach. "Community-based health insurance and access to maternal health services: evidence from three West African countries." Social science & medicine 66.12 (2008): 2460-2473. |
|  |  |  | - Data was collected using a questionnaire that was administered to 971 respondents in two communities selected by simple random sampling. Data analysis examined socio-economic status (SES) differences in enrolment levels, utilisation, willingness to renew registration and payments.  Enrolment level was 15.5% in the non-successful community and 48.4% in the successful community (p < 0.0001). However, there was no inequity in enrolment, willingness to renew registration and utilisation of services. Equal amounts of money were paid as registration fee and premium by all SES quartiles. There were no exemptions and no subsidies. | Onwujekwe, Obinna, et al. "Is community-based health insurance an equitable strategy for paying for healthcare? Experiences from southeast Nigeria." Health policy 92.1 (2009): 96-102. |
|  |  |  | - Community health insurance schemes have been successful in increasing assisted-delivery rates for scheme members by 45% in Rwanda47 and 12% in The Gambia.48 A seven times higher rate of delivery was reported in scheme members compared with non-members in the Democratic Republic of the Congo. | Borghi, J. O., et al. "Mobilising financial resources for maternal health." The Lancet 368.9545 (2006): 1457-1465. |
|  |  |  | - Utilisation of maternal health services % antenatal and delivery services, were significantly better at the late intervention period when compared to the early intervention period. Quality of service from clients' perspective also showed significant improvement at the late intervention period. There was an overall greater availability of maternal health service equipments, drugs and consumables, and medical records in the health facility later during the scheme. | Adinma, E. D., B. A. Nwakoby, and B. D. Adinma. "Integrating maternal health services into a health insurance scheme: effect on healthcare delivery." Nigerian quarterly journal of hospital medicine 20.2 (2009): 86-93. |
| **Health workforce** | Inadequate number and deployment of skilled workers in district hospitals, rural and remote areas e.g.: paediatricians and nurses | ***Expand the availability of health care workers*** *in all areas through the recruitment of local staff residing in the community (IND)* |  |  |
|  | Inadequate skilled workers able to manage newborn infections at the facility and community levels | ***Develop an electronic human resources training database*** *linked to the profile of health care workers to ensure all staff are trained (IND)* |  |  |
|  | Lack of job aids/charts on sepsis management; poor knowledge of existing guidelines in facilities |  |  |  |
|  | Poor health worker empowerment / motivation /incentives | ***Motivate of health providers through institutionalisation of incentives*** *to improve their retention in rural and hard to reach areas such as improved welfare packages or wages, hardship allowances, pay for performance (NGA, IND, KEN)* | - It was noted that financial incentives should be integrated with other incentives, particularly with regard to migration where it was concluded that financial incentives alone would not keep health workers from migrating. Nevertheless, Low salaries were found to be particularly de-motivating as health workers felt that their skills were not valued. - Career development was identified in 85% of the studies. Health workers were reluctant to work in rural areas as opportunities for career development were typically less than in urban areas [22]. The studies indicated that health workers take pride and are motivated when they feel they have the opportunity to progress. Job definition was also important, not only in terms of affecting general satisfaction and organisational commitment, but also for supervision and how staff assessed how they were getting along. Recognition and/or appreciation, either from managers, colleagues, or the community was a theme found in 70% studies. In some articles, recognition by the employer and community was cited as being one of the most important motivating factors for health workers. | Willis-Shattuck, Mischa, et al. "Motivation and retention of health workers in developing countries: a systematic review." BMC Health Services Research 8.1 (2008): 247. |
|  | Insufficient competency-based training; weak supervision and mentoring and post-training support | ***Conduct large scale pre-service and in-service trainings of health care workers in newborn care*** *including management of neonatal infections; Enhance the quality of trainings provided through supervision, mentoring and certification systems; involve private medical colleges and institutions; Train community-based health care workers in newborn care (BGD, NGA, KEN, VTN, IND, DRC, CAM)* | - The neonatal quality improvement initiative provided focused clinical training to 278 participants. A comparison of pre- and post-training test results demonstrates significant improvement in provider knowledge (73% vs. 89% correct, P < 0.001), with even greater improvement among trainees receiving recurrent refresher training (86% vs. 94% correct, P < 0.001). | Brantuo, Mary NA, et al. "Evidence‐based training and mentorship combined with enhanced outcomes surveillance to address the leading causes of neonatal mortality at the district hospital level in Ghana." Tropical Medicine & International Health 19.4 (2014): 417-426. |
|  |  |  | - Studies consistently provided opinions from health workers who stated that their supervisor's management and leadership skills were inadequate and this led to de-motivation of the workforce. Skilled managers have the ability to motivate their employees, however often in resource-poor institutions, management roles are assigned to staff who are not adequately trained. Effective managers are also responsible for lobbying on behalf of health workers and without their commitment factors affecting health worker motivation will not be identified or addressed. - Training enables workers to take on more demanding duties and to achieve personal goals of professional advancement as well as allow them to cope better with the requirements of their job and was found to be especially important for young health professionals. | Willis-Shattuck, Mischa, et al. "Motivation and retention of health workers in developing countries: a systematic review." BMC Health Services Research 8.1 (2008): 247. |
| **Essential medical products and technologies** | Stock-outs/irregular supply equipment to manage sick newborns including injection materials and antibiotics | ***Implement policy****, especially essential medicine policy to ensure enlisted commodities and medicines are available and accessible at service delivery points (NGA)* | - Delhi took the lead in developing a comprehensive Drug Policy in 1994 and was the only Indian state to have such a comprehensive policy. The policy's main objective is to improve the availability and accessibility of quality essential drugs for all those in need. The average number of stock-out days for key drugs decreased from 110 to 24 in the 500-bedded non-teaching tertiary care hospital and from 43 to 31 in other tertiary care hospitals. The incidence of stock-outs for Child-Health commodities decreased from 22.9% to 9.4% and for family planning-commodities, from 8.2% to 4.1% | Chaudhury, R. Roy, et al. "Quality medicines for the poor: experience of the Delhi programme on rational use of drugs." Health Policy and Planning 20.2 (2005): 124-136. |
|  |  |  |  |  |
|  |  | ***Strengthen the national procurement and supply system****: Forecast adequate amount of injectable antibiotics according to the needs for treatment and based on the buffer to be kept (NGA, VTN, IND); Streamline the procurement process including fast tracking of essential commodities (KEN, COD, NGA, CAM); Special equipment like, CPAP, portable x-ray, USG and arterial blood gas analyser should made available for tertiary care centres (IND)* | - To prevent stockouts and to increase the availability of health commodities nationwide, the USAID \| DELIVER PROJECT partnered with the Government of Nepal/Ministry of Health and Population (Logistics Management Division/Department of Health Services [LMD/DOHS]) and others to develop a series of logistics training programs. - The result is that stockout rates in Nepal have dropped. Family planning commodity stockouts are down by 50 percent—to only 4.1 percent. Even more dramatic are the levels of maternal and child health commodities; they have decreased from 22.9 percent to only 9.4 percent in three years. Now more women and children have access to the care they need. | USAID: DELIVER Project (2009) After Receiving USAID/DELIVER Project Logistics Training, Access to Health Products Improces in Nepal. Newsbrief, July, 2009. http://deliver.jsi.com/dhome/resources/publications/logisticsbriefs. |
|  |  |  | - The study compared three different groups of health facilities: those that received supervision for either use of STG (n = 23) or stock management (n = 21) - each facility acting as control for the other area of supervision - and a comparison group of facilities which received no supervision (n = 18). On-the-spot supervision by a specially trained pharmacy staff, based around identified deficiencies, took place at the start of the study and 3 months later. The evaluation compared performance on a variety of drug management indicators at baseline and 6-8 months after the second supervisory visit. There was a 13% improvement in availability in intervention group vs. control (STG) group (P = 0.284), 10% improvement vs. no intervention (P = 0.443). Thus no significant difference in actual availability. 14% improvement in overall stock management indicators vs. control (STG) group (P 0.001), 14% improvement vs. no intervention (P < 0.001). | Trap, Birna, et al. "The impact of supervision on stock management and adherence to treatment guidelines: a randomised controlled trial." Health policy and planning 16.3 (2001): 273-280. |
|  | Ineffective PSM system; Manufacturing gaps and inadequate local markets to facilitate the procurement and distribution of antibiotics | ***Establish quality control mechanisms****: Auditing of medical stores; facility assessments of those reporting frequent stock-outs; assessment of the quality of antibiotics in the bidding process; ensure the delivery of quality products (VTN, IND, PAK); Develop an electronic logistic management system (NGA, IND);* | - At a tertiary children's hospital, QI methods were used to rapidly implement the Pediatric Infectious Disease Society/Infectious Disease Society of America guideline recommendations for appropriate first-line antibiotic therapy in children with CAP. QI interventions focused on 4 key drivers and were tested separately in the emergency department and on the hospital medicine resident teams, using multiple plan-do-study-act cycles. Medical records of eligible patients were reviewed weekly to determine the success of prescribing recommended antibiotic therapy. The impact of these interventions on our outcome was tracked over time on run charts. - Appropriate first-line antibiotic prescribing for children admitted with the diagnosis of CAP increased in the emergency department from a median baseline of 0% to 100% and on the hospital medicine resident teams from 30% to 100% within 6 months of introducing the guidelines locally at Cincinnati Children's Hospital Medical Center and has been sustained for 3 months | Ambroggio L, Thomson J, Murtagh Kurowski E, Courter J, Statile A, Graham C, Sheehan B, Iyer S, Shah SS, White CM. Quality improvement methods increase appropriate antibiotic prescribing for childhood pneumonia. Pediatrics. 2013 May;131(5):e1623-31. doi: 10.1542/peds.2012-2635. Epub 2013 Apr 15. |
| **Health service delivery** | Poor service availability: lack of infrastructures for sick newborns; no space leading to short hospital stay limiting postnatal care at 48h | ***Scale-up newborn care at the lower level of care****: Strengthen the provision of newborn care, especially sepsis case management at primary health care level, institutionalise home visits / domiciliary care by trained personnel; Expand mobile outreach MNH services to make newborn services closer to the community (KEN, NGA, PAK, CAM, COD)* | - Home-based newborn care consisting of sepsis management, supportive care of low birthweight newborn babies, asphyxia management, primary prevention, health education and training of traditional birth attendants. Built upon a pre-existing community mobilisation programme. - Newborn mortality rate in the control area showed an increase from 58 in 1993–1995 to 64 in 2001–2003. The rate fell by 70% (95% CI 59–81%) compared with the control area. Early newborn mortality rate decreased by 64% and late newborn mortality rate by 80%. Still birth rate decreased 49% and the perinatal mortality rate by 56%. Newborn mortality rate did not change, and the infant mortality rate decreased by 57%, (95% CI 46–68%). Cause-specific newborn mortality rate (1995–1996 vs. 2001–2003) for sepsis decreased by 90%, for asphyxia by 53%, and for prematurity by 38% | AT Bang, HM Reddy, MD Deshmukh, SB Baitule, RA Bang Effect of home-based neonatal care and management of sepsis on neonatal mortality: field trial in rural India Lancet, 354 (1999), pp. 1955–1961  AT Bang, HM Reddy, MD Deshmukh, SB Baitule, RA Bang Neonatal and infant mortality in the ten years (1993 to 2003) of the Gadchiroli field trial: effect of home-based neonatal care J Perinatol, 25 (Suppl 1) (2005), pp. S92–S10 |
|  |  |  | - Female community workers doing home care visits (1 per 4000) and community mobilisers running health education groups every 4 months vs. control comparison areas. Newborn mortality rate was reduced in the home-care arm by 34% (adjusted relative risk 0·66; 95% CI 0·47–0·93) during the last 6 months of the trial vs. that in the comparison arm. No mortality reduction was noted in the community-care arm (0.95; 0·69–1·31). | AH Baqui, S El-Arifeen, GL Darmstadt, et al. Projahnmo Study Group. Effect of community-based newborn-care intervention package implemented through two service-delivery strategies in Sylhet district, Bangladesh: a cluster-randomised controlled trial. Lancet, 371 (2008), pp. 1936–1944 |
|  | Ineffective referral system to transfer sick newborns from communities to secondary and tertiary hospitals | ***Strengthen two way referral system*** *for sick newborns (IND, KEN, VTN, COD, NGA)* |  |  |
|  | Community-based support for sepsis case management not defined/ adequately integrated in health clinics | ***Ensure post-discharge counselling on danger signs*** *for newborns (IND)* | - This study examined the effect of a job aids-focused intervention on quality of facility-based postnatal counselling, and whether increased communication improved in-hospital newborn care and maternal knowledge of home practices and danger signs requiring urgent care. Using a pre-post randomised design, data were drawn from direct observations and interviews with 411 mother-newborn pairs. Multi-level regression models with difference-in-differences analyses estimated the intervention's relative effect, adjusting for changes in the comparison arm. The mean percent of recommended messages provided to recently-delivered women significantly improved in the intervention arm as compared to the control (difference-in-differences [∆i - ∆c] +30.9, 95 % confidence interval (CI) 19.3, 42.5), and the proportion of newborns thermally protected within the first hour (∆i - ∆c +33.7, 95 % CI 19.0, 48.4) and delayed for bathing (∆i - ∆c +23.9, 95 % CI 9.4, 38.4) significantly increased. No significant changes were observed in early breastfeeding (∆i - ∆c +6.8, 95 % CI -2.8, 16.4) which was nearly universal. Omitting traditional umbilical cord substances rose slightly, but was insignificant (∆i - ∆c +8.5, 95 % CI -2.8, 19.9). The proportion of mothers with correct knowledge of maternal (∆i - ∆c +27.8, 95 % CI 11.0, 44.6) and newborn (∆i - ∆c +40.3, 95 % CI 22.2, 58.4) danger signs grew substantially, as did awareness of several home-care practices (∆i - ∆c +26.0, 95 % CI 7.7, 44.3). | Jennings, L., et al. "Use of Job Aids to Improve Facility-Based Postnatal Counseling and Care in Rural Benin." Maternal and child health journal (2014): 1-9. |
|  |  |  |  |  |
|  | No review of quality of care checklists; lack of SOPs/job aids; Inadequate system to ensure adherence to guidelines in countries where available | ***Establish quality assurance mechanisms****: a quality assurance cell at the state/national level with regular quality assessments with emphasis on supportive supervision and mentoring by medical colleges and private hospitals (IND); national scale-up of clinical audits and perinatal death reviews (BGD, CAM); Periodic critical review of appropriate management of newborn infections (NGA); Regular review and dissemination of quality of care check lists (NGA)* | - Ten low-quality evaluations with mortality outcome data were identified. Meta-analysis of 7 before-and-after studies indicated a reduction in perinatal mortality of 30% (95% confidence interval, 21%–38%) after introduction of perinatal audit. | Pattinson, Robert, et al. "Perinatal mortality audit: counting, accountability, and overcoming challenges in scaling up in low-and middle-income countries." International Journal of Gynecology & Obstetrics 107 (2009): S113-S122. |
| **Health management information system** | Indicator on neonatal infection not captured in national HMIS; No neonatal registers/ repository of health information | ***Strengthen data collection and reporting for newborn care:*** *disaggregate the data in the health management information system to include newborn health interventions, especially management of severe neonatal infections; add community data and postnatal consultations; develop neonatal registers; and set up a monitoring system for hospital infection prevention (COD, KEN, CAM, VTN, PAK)* |  |  |
|  |  |  |  |  |
|  |  | ***Develop electronic reporting system:*** *software apps for record keeping on newborn interventions including neonatal sepsis management and establishing linkages from facility to community (IND)* |  |  |
|  |  | ***Analyse and use data on management of neonatal infections*** *to inform performance review meetings and for quality improvement processes (PAK)* |  |  |
|  | Lack of review process to improve sepsis case management; no integration of neonatal infections in perinatal audits | ***Enhance research in newborn care:*** *conduct bacterial surveillance and antibiotics resistance studies; conduct newborn survival analysis to inform program managers (NGA, VTN)* |  |  |
|  | Lack of awareness and knowledge on danger signs for neonatal infections | ***Development/review of local IEC materials****: emphasise benefits of newborn care services, mainly within the first week of life (NGA)* |  |  |
|  |  |  |  |  |
|  |  | ***Strengthen community-based activities****: education on hand washing and personal hygiene, behaviour change communication activities for educating caretakers on identification and prompt care seeking and to tackle harmful cultural beliefs, awareness campaigns using multiple channels to increase knowledge, demand for postnatal and community-based newborn care, and empower women (BGD, NGA, VTN, PAK, KEN)* | - A control group, received the usual services of governmental and non-governmental organisations in the area; an intervention group, which received a preventive package of interventions for essential newborn care (birth preparedness, clean delivery and cord care, thermal care [including skin-to-skin care], breastfeeding promotion, and danger sign recognition); or another intervention group, which received the package of essential newborn care plus use of a liquid crystal hypothermia indicator (ThermoSpot). - Improvements in birth preparedness, hygienic delivery, thermal care (including skin-to-skin care), umbilical cord care, skin care, and breastfeeding were seen in intervention arms. There was little change in care-seeking. Compared with controls, neonatal mortality rate was reduced by 54% in the essential newborn-care intervention (rate ratio 0·46 [95% CI 0·35–0·60], p<0·0001) and by 52% in the essential newborn care plus ThermoSpot arm (0·48 [95% CI 0·35–0·66], p<0·0001). | Kumar, Vishwajeet, et al. "Effect of community-based behaviour change management on neonatal mortality in Shivgarh, Uttar Pradesh, India: a cluster-randomised controlled trial." The Lancet 372.9644 (2008): 1151-1162. |
|  |  |  |  |  |
|  |  |  | - Nine distinct studies contributing data for community-based management of neonatal pneumonia and sepsis were identified. In a pooled analysis of 5 controlled trials of community-based management of neonatal pneumonia (4 using cotrimoxazole, 1 ampicillin, or penicillin), all-cause neonatal mortality showed 27% [95% confidence interval (CI): 18%-35%] reduction and pneumonia-specific mortality, 42% (95% CI: 22%-57%). Substantial reductions in neonatal mortality have been demonstrated in a non-randomised controlled study in rural India (62% reduction, P < 0.001) and in a cluster randomised trial in rural Bangladesh (34% reduction, 95% CI: 7%-53%). Reduced case fatalities (0%-3%) with community-based management of neonatal sepsis were observed in 2 small uncontrolled studies from India and Guatemala and a recent randomised trial from Pakistan. | Bhutta, Zulfiqar A., et al. "Management of newborn infections in primary care settings: a review of the evidence and implications for policy?." The Pediatric infectious disease journal 28.1 (2009): S22-S30. |
| **Community ownership and participation** | Low community engagement and mobilisation including male partners in maternal and newborn care | ***Engage male partners in MNH care****: Encourage male participation in ANC, labour, delivery and post natal visit (IND, NGA, COD, CAM)* |  |  |
|  | Barriers to access /use care (eg. Poor care seeking for newborn care, lack of transport, long distance to facility) |  |  |  |
|  | Socio-cultural factors (misconceptions, seclusion of newborns, topical application on cord, etc.) |  |  |  |
|  | Poor compliance in receiving full treatment for neonatal infections |  |  |  |
